# *O*^6^-methylguanine DNA methyltransferase and glucose transporter 2 in foregut and hindgut gastrointestinal neuroendocrine neoplasms

**DOI:** 10.1186/s12885-020-07579-6

**Published:** 2020-12-07

**Authors:** Hirofumi Watanabe, Yuto Yamazaki, Fumiyoshi Fujishima, Komoto Izumi, Masayuki Imamura, Susumu Hijioka, Kazuhiro Toriyama, Yasushi Yatabe, Atsushi Kudo, Fuyuhiko Motoi, Michiaki Unno, Hironobu Sasano

**Affiliations:** 1 Department of Pathology, Tohoku University, Graduate School of Medicine, Sendai, Miyagi 980-8575 Japan; 2 Department of Surgery, Kansai Electric Power Hospital, Osaka, 553-0003 Japan; 3 Kansai Electric Power Medical Research Institute, Osaka, 553-0003 Japan; 4 Department of Hepatobiliary and Pancreatic Oncology, National Cancer Center, Tokyo, 104-0045 Japan; 5 Department of Pathology and Molecular Diagnostics, Aichi Cancer Center Hospital, Nagoya, Aichi 464-0021 Japan; 6 Department of Pathology and Clinical Laboratories, National Cancer Center, Tokyo, Japan; 7 Department of Hepato-Biliary-Pancreatic Surgery, Graduate School of Medicine, Tokyo Medical and Dental University, Tokyo, 113-0034 Japan; 8 Department of Surgery I, Yamagata University Graduate School of Medical Science, Yamagata, 990-9585 Japan; 9 Department of Surgery, Tohoku University, Graduate School of Medicine, Sendai, Miyagi 980-8575 Japan

**Keywords:** Neuroendocrine neoplasm, *O*^6^-methylguanine DNA methyltransferase, Glucose transporter 2, Immunohistochemistry

## Abstract

**Background:**

Streptozocin (STZ) is used for treating both pancreatic (PanNET) and gastrointestinal (GI-NET) neuroendocrine tumors but its therapeutic efficacy is relatively low in GI-NETs. Therefore, it has become pivotal to select GI-NET patients who could benefit from STZ treatment. STZ is transported via the glucose transporter 2 (GLUT2) into the cells and the loss of O6-methylguanine DNA methyltransferase (MGMT) also increases its therapeutic efficacy. Therefore, GLUT2 high and MGMT low status could be the surrogate markers of STZ.

**Methods:**

In this study, we examined the MGMT and GLUT2 status in gastrointestinal neuroendocrine neoplasm (NEN). We studied 84 NEN cases: 33 foregut and 37 hindgut GI-NETs and 14 gastrointestinal neuroendocrine carcinomas (GI-NECs).

**Results:**

In GI-NETs, MGMT scores of ≥2 and ≥ 3 were 77% (54/70) and 56% (39/70), respectively, and GLUT2 scores of ≥4 and ≥ 6 were 30% (21/70) and 4.3% (3/70), respectively. Methylation-specific polymerase chain reaction revealed that MGMT promoter methylation was detected only in 2/14 GI-NECs but none of the included GI-NETs. GLUT2 (GLUT2 score) and MGMT immunoreactivity (MGMT and H-scores) were both significantly correlated with Ki-67 labeling index (GLUT2 score: *P* = 0.0045, ρ = − 0.4570; MGMT score: *P* = 0.0064, ρ = − 0.4399; H-score: *P* = 0.0110, ρ = − 0.4135) and MGMT immunoreactivity were significantly correlated with GLUT2 immunoreactivity (MGMT score: *P* = 0.0198; H-score, *P* = 0.0004, ρ = 0.5483) in hindgut NETs, but not in foregut NETs. However, discrepancies from the above correlation between GLUT2 and MGMT immunoreactivity were detected in several GI-NET cases which could be potential candidates for STZ therapy.

**Conclusion:**

The evaluation of MGMT and GLUT2 status could provide an important information in planning STZ therapy in GI-NET patients.

**Supplementary Information:**

The online version contains supplementary material available at 10.1186/s12885-020-07579-6.

## Background

Neuroendocrine tumors (NETs) are relatively rare tumors that comprise approximately 0.5% of all newly diagnosed human malignancies, but their incidence has recently increased over the years [1]. The gastrointestinal (GI) tract is one of the most frequent primary sites of NETs, and 12–22% of patients with NETs harbor metastatic lesions at the time of initial clinical diagnosis [1]. Therefore, metastasis is, by no means, rare in NETs but surrogate markers of systemic therapy have not necessarily been established in GI-NET cases with metastasis [2]. In addition, the clinicopathological features of GI-NETs differ according to their embryonic origins—fore-, mid-, and hindgut—and therefore the origins of NETs should also be considered when deciding their treatment strategy of the patients [3].

Streptozocin (STZ) is a DNA-alkylating agent that exerts its therapeutic effects through promoting apoptosis in tumor cells [4]. In patients with NETs, a combination of 5-fluorouracil (FU) plus STZ was reported to significantly improve the median survival of the patients with pancreatic, GI, and pulmonary advanced carcinoid tumors [5]. Therefore, STZ is currently used as a cytotoxic agent for treating pancreatic NET (PanNETs) and GI-NETs. However, its response rate is 40.0% for pancreaticoduodenal NETs and only 25.0% for GI-NETs [6]. In addition, significant improvement of progression-free survival of STZ has not necessarily been established in patients with GI-NETs compared to those with PanNETs, especially in foregut and hindgut NETs [5, 7, 8]. Temozolomide, another alkylating agent [9], has been used for some patients with both PanNETs or GI-NETs [10], but its clinical efficacy has not yet been established in GI-NETs [11, 12]. In addition, various side effects have been reported when using alkylating agents, including STZ, for treating the patients with NETs [13, 14]. Therefore, it has become crucial to select the patients with GI-NETs who could benefit from this treatment of alkylating agent.

The therapeutic efficacy of STZ has been reported to be influenced by the status of both glucose transporter 2 (GLUT2) and *O*^6^-methylguanine-DNA methyltransferase (MGMT) in tumor cells [15, 16]. STZ is actively transported to β cells via GLUT2 in the pancreas [14]. GLUT2 is a low-affinity glucose transporter expressed in pancreatic islet cells and involved in insulin secretion [17]. In GI tract, GLUT2 is expressed in enteroendocrine L-cells in both small and large intestines and epithelial cells in small intestine [18, 19]. MGMT is an enzyme that repairs DNA modifications, consequently preventing carcinogenesis [20]. Because of its promoter methylation, the loss of MGMT was also reported to be correlated with increased frequency of p53 point mutations in astrocytoma [21] and associated with adverse clinical outcome in lung or biliary tract cancer patients [20, 22]. Therefore, among the patients with neuroendocrine neoplasms (NENs), MGMT promoter methylation is postulated to be detected more frequently in high grade NENs than low or intermediate grade NENs. In addition, the loss of MGMT in tumor cells, including pancreatic neuroendocrine neoplasms [23], was reported to increase the therapeutic efficacy of DNA-alkylating agents, including STZ [20]. Therefore, STZ could be effective on MGMT low and GLUT-2 high NEN patients. However, in-depth information is not necessarily available regarding GLUT2 and MGMT status, which could influence therapeutic efficacy, especially in GI-NEN patients, some of whom could possibly benefit from STZ therapy [6].

Therefore, in this study, we examined MGMT and GLUT2 status using immunohistochemistry (IHC) and methylation analysis in cases of MGMT in order to explore their clinicopathological significance in GI-NEN patients.

## Methods

### GI-NEN cases

Surgical specimens of GI-NENs (Supplementary Table 1) and their metastatic lesions (lymph nodes and liver, (Supplementary Table 2) from 2002 to 2019 were retrieved from surgical pathology files at Tohoku University Hospital (Sendai, Japan), Aichi Prefectural Cancer Center Hospital (Nagoya, Japan), Noe Hospital (Osaka, Japan), Tokyo Medical and Dental University Hospital (Tokyo, Japan), and Kansai Electric Power Hospital (Osaka, Japan). 10% formalin fixed and paraffin embedded tissue blocks (FFPE) were available for this study in the specimens from Tohoku University Hospital, but only unstained serial tissue slides for immunohistochemistry and hematoxylin and eosin stain were available in those from other institutions above. The clinicopathological features of these NEN cases were summarized in Table 1. The research protocol of this study was approved by the institutional review boards of Tohoku University Graduate School of Medicine (2020-1-7) and the institutions above.

**Table 1:** Summary of results and clinicopathological characteristics of GI-NEN cases examined in this study

Patient characteristics of neuroendocrine tumor	
Total number	70
Sex, male/female	42/28
Median age in years (range)	60 (33–82)
Grade (WHO 2019)	NET-G1, *n* = 49; NET-G2, *n* = 19; NET-G3, *n* = 2
Primary lesion of NET	Foregut, *n* = 33; Hindgut, *n* = 37
Case of lymph node metastasis in NET G1	Metastatic (pathologically), *n* = 5; Not detected (clinically), *n* = 20;Not detected (pathologically), *n* = 10; Unclarified, *n* = 14
MGMT score	Score0, n = 1; Score1, *n* = 15; Score2, *n* = 15; Score3, *n* = 39
GLUT2 score	Score0, *n* = 21; Score1, n = 10; Score2, *n* = 18; Score4, *n* = 18; Score6, *n* = 3
MGMT methylation specific PCR	Negative, *n* = 35; Positive, *n* = 0; Not examined, *n* = 35
Median Ki-67 labeling index^a^ (range)	1.89 (0.33–48.3)
Patient characteristics of neuroendocrine carcinoma	
Total number	14
Sex, male/female	10/4
Median age in years (range)	70 (60–86)
Primary lesion of NEC	Foregut, *n* = 10; Hindgut, *n* = 4
MGMT score	Score0, *n* = 1; Score1, *n* = 0; Score2, *n* = 4; Score3, *n* = 9
MGMT methylation specific PCR	Negative, *n* = 12; Positive, *n* = 2

^a^Round the fourth digit

Serial tissue sections of FFPE specimens were used for subsequent analyses. The cases included 70 GI-NET and 14 GI-NEC. The GI-NET cases were tentatively classified into foregut (*n* = 33) and hindgut (*n* = 37) GI-NETs according to their primary sites. These cases were further classified into G1 (*n* = 49), G2 (*n* = 19), and G3 (*n* = 2) tumors based on the grading criteria of the 2019 WHO Classification [24].

### Immunohistochemistry/IHC

After carefully reviewing the available hematoxylin and eosin (H&E)-stained slides microscopically, one representative section including the tumor area in its greatest dimensions was selected in each case. Serial tissue sections were prepared at 3-μm thickness. The IHC protocols were summarized in Table 2. Representative images of IHC positive control were illustrated in Supplementary Figure 1.

**Table 2:** Summary of immunohistochemistry procedures used in this study

Antibody	Antigen retrieval treatment	Supplier	Dilution	Clone	Control
Ki-67	PT Link (97 °C, 20 min) Target Retrieval Solution High PH	DAKO, Denmark	Ready to use	MIB-1	Epithelial cell
GLUT2	AC (121 °C, 5 min), pH 6.0	proteintech, USA	1:500	polyclonal	Islet of langerhans
MGMT	AC (121 °C, 5 min), pH 6.0	Millipore, USA	1:200	MT3.1	Vascular endothelial cell

Immunostained slides were digitally scanned using Nanozoomer S360 (Hamamatsu Photonics, Shizuoka, Japan) for the subsequent imaging analysis.

### Evaluation of Ki-67 labeling index

**Fig. 1:**
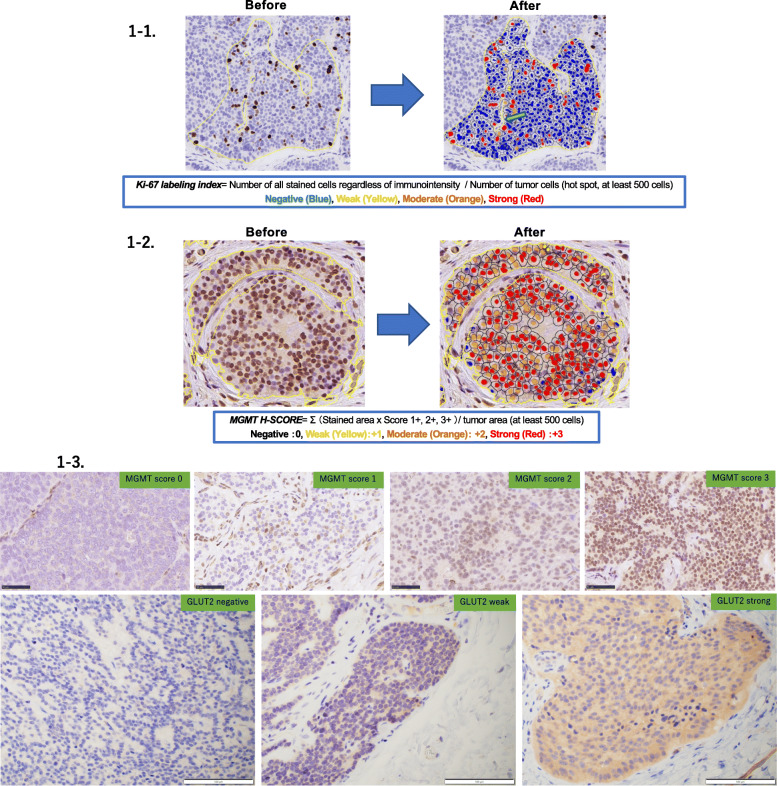
Representative immunohistochemistry illustrations of MGMT, GLUT2, and Ki-67. **1-1** Representative illustrations before and after digital image analyses of Ki-67 LI. We analyzed nuclear immunoreactivity according to the gradients of brown color (3,3-diaminobenzidine [DAB]) spectrum immunointensity. Tumor cells with blue nuclei were negative, whereas those with yellow, orange, and red nuclei were positive for Ki-67 immunoreactivity. The arrow indicated intratumoral vessels excluded. **1-2** Representative illustrations before and after digital image analyses of MGMT immunoreactivity. MGMT was analyzed only in nuclei according to the gradients of brown color (3,3-diaminobenzidine [DAB]) spectrum intensity. The relative immunointensity of the gradient was evaluated as follows: 0, negative (blue); + 1, weak (yellow); + 2, moderate (orange); + 3, strong (red). **1-3** MGMT and GLUT2 scores

The Ki-67 labeling index (LI) was determined according to the counting method defined by WHO in 2019 [24, 25], using the HALO image analysis software (Indica Laboratories, Corrales, New Mexico, USA) with the CytoNuclear IHC v1.6 algorithm module (Supplementary Figures 2, 3, 4). We performed imaging analysis, according to a previously reported study using this digital data to obtain the Ki-67 LI [26]. We analyzed nuclear immunoreactivity according to the gradients of brown color (3,3-diaminobenzidine [DAB]) spectrum intensity. Tumor cells with blue nuclei were negative, whereas cells with yellow (weak intensity), orange (moderate intensity), and red (strong intensity) nuclei were positive for Ki-67 immunoreactivity. The labeling index or LI was calculated based on the following formula: Number of all stained cells regardless of immunointensity/number of tumor cells (hot spot, at least 500 cells). Representative images obtained before and after the analysis were illustrated in Fig. 1-1.

### Evaluation of MGMT immunohistoreactivity

MGMT immunoreactivity was detected in the nuclei and evaluated independently using two different scoring systems, i.e., MGMT score and H-score.

**Fig. 2:**
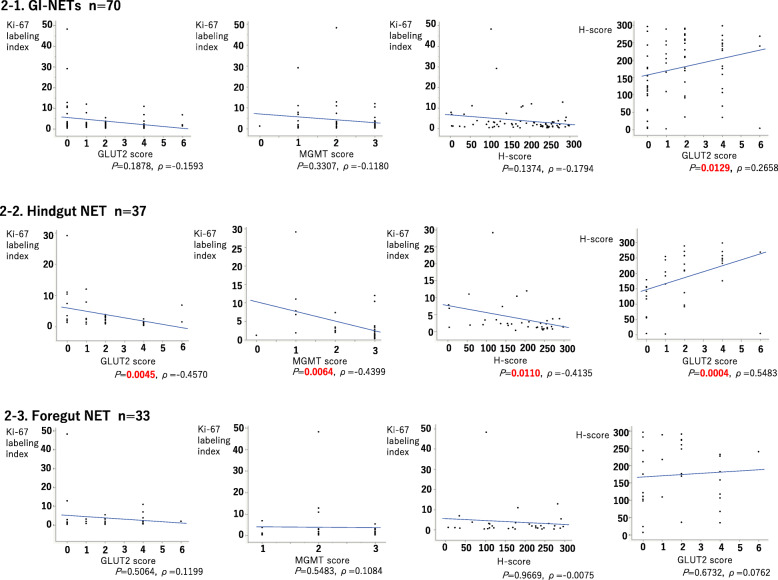
Correlation among MGMT and GLUT2 scores and Ki-67 LI in foregut and hindgut NETs. **2-1** Results of MGMT and GLUT2 immunoreactivity in all the GI-NET cases examined. There was a significant positive correlation between GLUT2 and H-scores, but not between the GLUT2 score, MGMT score or H-score and Ki-67 LI. **2-2** Results of MGMT and GLUT2 immunoreactivity of hindgut NET cases. There was a significant inverse correlation between Ki-67 LI and GLUT2, MGMT, and H-scores. There was a significant positive correlation between the GLUT2 and H-score. **2-3** Results of MGMT and GLUT2 immunoreactivity of foregut NET cases. There were no significant correlations between the GLUT2, MGMT, or H-scores and Ki-67 LI

The H-score was obtained using the HALO image analysis software with the CytoNuclear IHC v1.6 algorithm module. Parameter of “Cell detection” (nuclear contrast, optical density, size, and shape) and the thresholds of immunoreactivity in each section were set according to those previously reported [26]. The image analysis was performed by a single observer in the average areas. The average area was randomly selected and analyzed by counting more than 500 cells. All the parameters were set individually in each case. In the selected annotation areas, the HALO software automatically calculated the number of positive cells with weak, moderate, and strong immunoreactivity among the total cells. Representative images of analytical procedures were illustrated in Fig. 1-2. The H-score was subsequently calculated based on the following formula: Σ (individual gradients of the positive tumor cells/all tumor cells × Score 1+, 2+, 3+).

**Fig. 3:**
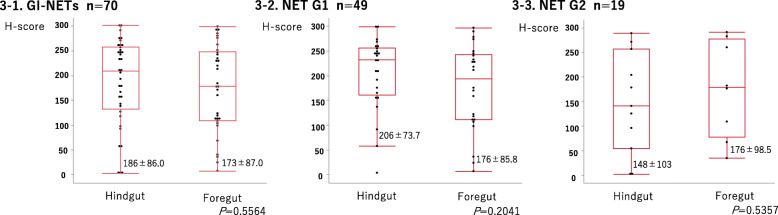
Comparison of MGMT immunoreactivity with relation to individual histological grades of foregut and hindgut NETs. **3-1** Comparison of H-scores between foregut and hindgut GI-NETs. The H-scores were not significantly different between foregut and hindgut NETs. **3-2** Comparison of H-scores between foregut and hindgut G1 GI-NETs. H-scores were not significantly different between foregut and hindgut G1 NETs. **3-3** Comparison of H-scores between foregut and hindgut G2 GI-NETs. H-scores were not significantly different between foregut and hindgut G2 NETs. Data are expressed as mean ± SD (round the fourth digit)

The MGMT score was obtained by microscopic and manual/eyeball analysis performed by three of the authors (H.W., F.F., and H.S.). The MGMT score was determined according to a previously reported study [27], incorporating the proportion of positive nuclear immunoreactivity in tumor cells as follows: score 0, absence of immunoreactivity; score 1, nuclear immunoreactivity in less than 20% tumor cells; score 2, nuclear immunoreactivity in greater than 20% but less than 50% tumor cells; and score 3, immunoreactivity in greater than 50% tumor cells. Representative images of MGMT scores were illustrated in Fig. 1-3.

### Evaluation of GLUT2 immunoreactivity

We evaluated the status of GLUT2 immunoreactivity with a semiquantitative scoring system assessing both the proportion and relative immunointensity according to Kaemmerer et al. [28], using microscopic and manual/eyeball analysis performed by three of the authors (H.W., F.F., and H.S.). The proportion of immunopositive cells was tentatively classified into three different categories: proportion score 0, completely negative; 1, 1–50% cells positive; and 2, 51–100% cells positive. The relative immunointensity of positive cells was further sub-classified into 4 categories: intensity score 0, completely negative; 1, weak; 2, weak and strong (the tumor area presenting weak and strong immunointensity was respectively and simultaneously detected in more than 10% of positive tumor area); and 3, strong. Representative images for the GLUT2 intensity score were illustrated in Fig. 1-3, and the GLUT2 score was subsequently calculated using the following formula: proportion score × intensity score.

### Evaluation of MGMT promoter methylation with methylation-specific real-time PCR

Forty-nine FFPE specimens of GI-NENs including 35 NETs and 14 NECs were retrieved from the pathology files of Tohoku University Hospital. Serial tissue sections at 10-μm thickness were prepared following the macro-dissection of relevant tumor areas. DNA was extracted from these specimens above using the Cobas DNA preparation kit (Roche, Mannheim, Germany). Tumor DNA was treated with bisulfite using the MethylEasy™ Xceed Rapid DNA Bisulphite Modification Kit (Takara Bio Inc., Shiga, Japan), following the manufacturer’s instructions. 1.0 μl of tumor DNA template (20 ng/μl) treated with bisulfite was mixed with 10.0 μl of LightCycler® 480 Probes Mater (Roche Diagnostics, Mannheim, Germany), 1.0 μl of Forward Primer, 1.0 μl of Reverse Primer, 1.0 μl of TaqMan Probe and 6.0 μl of H_2_0. MGMT promoter methylation was evaluated by methylation-specific real-time PCR in a LightCycler 480 Real-Time PCR System (Roche) for preincubation (10 min, 95 °C), amplification (15 s, 95 °C and 1 min, 60 °C) 50 cycles and cooling (30 s, 40 °C), with reference to the method reported by Sonoda et al. and Kitange et al. [27, 29]. We used β-actin as Housekeeping gene. The primer sequences (obtained from Nihon Gene Research Laboratories INC, Sendai, Japan), 5′-TTCGCGGTGCGTATCGT-3′ (forward) and 5′-CACTCTTCCGAAAACGAAACGA-3′ (reverse), were used for the methylation reaction and 5′-TTTTATTTAGAGTGTAGGTGTGTGGAGATTTT-3′ (forward) and 5′-CAAAAACAAAAACCTAACCCCTAAACCT-3′ (reverse) for β-actin. The probe sequence 5′-FAM-ACACTCACCAAATCGC-MGB-3′ (TaqMan® MGB, Thermo Fisher Scientific, Tokyo, Japan) was used for the methylation reaction and 5′-FAM-CCCACCCTCTAAAACT-MGB-3′ (TaqMan® MGB) for β-actin. Cp Genome Universal Methylated DNA (Merck, Darmstadt, Germany) was used as MGMT methylation control DNA.

### Statistical analysis

The differences of MGMT and GLUT2 immunoreactivity were analyzed using χ^2^ test or Mann-Whitney’s *U* test. The correlation between Ki-67 LI and MGMT (H- and MGMT scores) and GLUT2 (GLUT2 score) immunoreactivity was analyzed by Spearman’s test. *P* values of < 0.05 were considered significant. The JMP Pro ver.14.3.0 software (SAS Institute, Inc., Cary, NC, USA) was used for statistical analysis.

## Results

### Correlation among MGMT and/or GLUT2 immunoreactivity and Ki-67 LI in foregut and hindgut NETs

Results of MGMT score, H-score, GLUT2 score, and Ki-67 LI of GI-NETs were summarized in Table 3 and Fig. 2. MGMT (MGMT and H-scores) and GLUT2 immunoreactivity (GLUT2 score) were both significantly correlated with Ki-67 LI in hindgut NETs (GLUT2 score: *P* = 0.0045, ρ = − 0.4570; MGMT score: *P* = 0.0064, ρ = − 0.4399; H-score: *P* = 0.0110, ρ = − 0.4135) but not in foregut NETs (GLUT2 score: *P* = 0.5064, ρ = 0.1199; MGMT score: *P* = 0.5483, ρ = 0.1084; H-score: *P* = 0.9669, ρ = − 0.0075). MGMT and H-scores were both significantly correlated with GLUT2 scores in hindgut NETs (MGMT score: *P* = 0.0198; H-score, *P* = 0.0004, ρ =0.5483) but not in foregut NETs (MGMT score: *P* = 0.6265; H-score: *P* = 0.6732, ρ = 0.0762).

**Table 3:** Correlation among MGMT and GLUT2 immunoreactivity

	GLUT2 score/ MGMT score	score 0	score 1	score 2	score 3	*P* value
GI-NETs	Score 0	1	8	7	5	0.0591
	Score 1	0	1	2	7	
	Score 2	0	1	4	13	
	Score 4	0	4^b^	2	12	
	Score 6	0	1^b^	0	2	
Hindgut NET	Score 0	1	3	3	2	0.0198^a^
	Score 1	0	1	1	5	
	Score 2	0	0	2	8	
	Score 4	0	0	0	9	
	Score 6	0	1^b^	0	1	
Foregut NET	Score 0	0	5	4	3	0.6265
	Score 1	0	0	1	2	
	Score 2	0	1	2	5	
	Score 4	0	4^b^	2	3	
	Score 6	0	0	0	1	

^a^Statistical significance

^b^GI-NET cases which could be potential candidates for STZ therapy

### Comparison of MGMT and GLUT2 in foregut and hindgut NETs according to their histological grades

As summarized in Table 4 and Fig. 3, MGMT, H-, and GLUT2 scores were not significantly different between foregut and hindgut NETs (MGMT score: *P* = 0.0839; H-score: *P* = 0.5564; GLUT2-score: *P* = 0.7025). Upon individual analyses of NET G1 and G2 specimens, no significant differences were detected in GLUT2 score, H-score for NET G1/G2 specimens, and MGMT score for NET G2 specimens. In the case of the MGMT score in NET G1 specimens, significant differences were detected between foregut and hindgut NETs (MGMT score: *P* = 0.0109, H-score: *P* = 0.2041; GLUT2-score: *P* = 0.3211).

**Table 4:** Comparison of MGMT and GLUT2 score in foregut and hindgut NETs according to histological grades

	GLUT2 score	Foregut	Hindgut	*P* value
GI-NETs	Score 0	12	9	0.7025
	Score 1	3	7	
	Score 2	8	10	
	Score 4	9	9	
	Score 6	1	2	
NET G1	Score 0	10	4	0.3211
	Score 1	2	5	
	Score 2	5	6	
	Score 4	6	9	
	Score 6	1	1	
NET G2	Score 0	1	4	0.2290
	Score 1	1	2	
	Score 2	3	4	
	Score 4	3	0	
	Score 6	0	1	
	MGMT score	Foregut	Hindgut	*P* value
GI-NETs	Score 0	0	1	0.0839
	Score 1	10	5	
	Score 2	9	6	
	Score 3	14	25	
NET G1	Score 0	0	1	0.0109^a^
	Score 1	8	1	
	Score 2	5	3	
	Score 3	11	20	
NET G2	Score 0	0	0	1.000
	Score 1	2	3	
	Score 2	3	3	
	Score 3	3	5	

^a^Statistical significance

### Comparison of MGMT and GLUT2 scores of primary tumors with metastatic lesions

Ki-67 LIs and the MGMT and GLUT2 scores in Table 5 demonstrated that the MGMT or GLUT2 scores of metastatic lesions were lower than those of primary tumors in cases 1, 2, 4 and 6, respectively.

**Table 5:** Comparison of MGMT and GLUT2 scores of primary tumors with metastatic lesions

Case No.^a^	Primary site	Grade	Ki-67 labeling index	GLUT2 score	MGMT score
case 1	Stomach	G2	10.9264	4	2
	Lymph node	G2	3.10345	0	2
case 2	Duodenum	G2	3.52588	2	3
	Lymph node	G2	3.46821	2	3
	Liver	G2	5.02431	1	3
case 3	Stomach	G2	3.7929	4	1
	Lymph node	G2	4.47471	4	1
case 4	Rectum	G2	6.83572	6	1
	Lymph node	G3	31.5287	0	1
case 5	Rectum	G2	11.0251	0	1
	Liver	G2	4.13534	0	1
case 6	Rectum	G1	2	2	2
	Liver	G2	3.37972	0	1

^a^Patient characteristics of these cases: male, *n* = 3; female, *n* = 3 / Median age in years (range), 65.5 (41–67)

### Correlation between MGMT or GLUT2 status and lymph node metastases

**Fig. 4:**
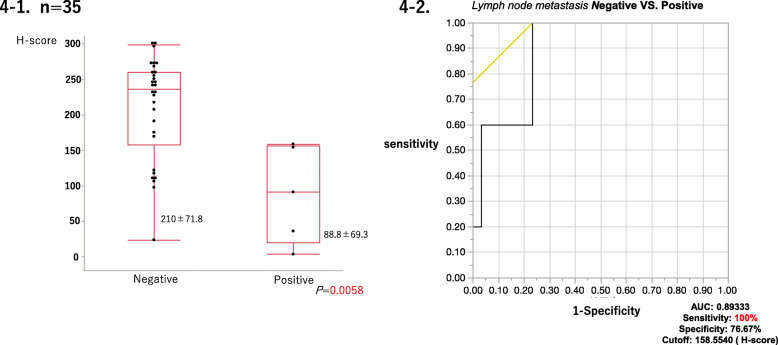
Comparison of MGMT immunoreactivity between the cases with and without lymph node metastasis in GI-NETs. **4-1** Among G1 GI-NET cases examined, MGMT immunoreactivity (H-scores) was significantly different between the cases with and without lymph node metastasis in GI-NETs. Data are expressed as mean ± SD (round the fourth digit). **4-2** Cases with lymph node metastasis were significantly correlated with H-Score by less than 158.5540 (area under the curve [AUC]: 0.89333; sensitivity: 100%; specificity: 76.67%)

Results summarized in Table 6 and Fig. 4 demonstrated that among 35 G1 GI-NET cases which were assessed clinical or pathological lymph node metastasis, MGMT status (MGMT and H-scores) was significantly different between the lymph node metastases positive (cN1 or pN1) and negative (neither cN1 nor pN1) cases (MGMT score: *P* = 0.0042; H-score: *P* = 0.0058). However, this difference was not statistically significant in GLUT2 status (GLUT2-score: *P* = 0.5465). In addition, the cases with lymph node metastasis were significantly correlated with H-Score by less than 158.5540 (area under the curve [AUC]: 0.89333; sensitivity: 100%; specificity: 76.67%).

**Table 6:** Correlation between MGMT or GLUT2 status and lymph node metastases

	Lymph node metastasis		
GLUT2 score	negative	positive	*P* value
Score 0	6	2	0.5465
Score 1	5	0	
Score 2	5	2	
Score 4	12	1	
Score 6	2	0	
MGMT score	negative	positive	*P* value
MGMT Score 0	0	1	0.0042^a^
MGMT Score 1	5	2	
MGMT Score 2	4	2	
MGMT Score 3	21	0	

^a^Statistical significance

### Comparison of MGMT immunoreactivity between NETs and NECs

**Fig. 5:**
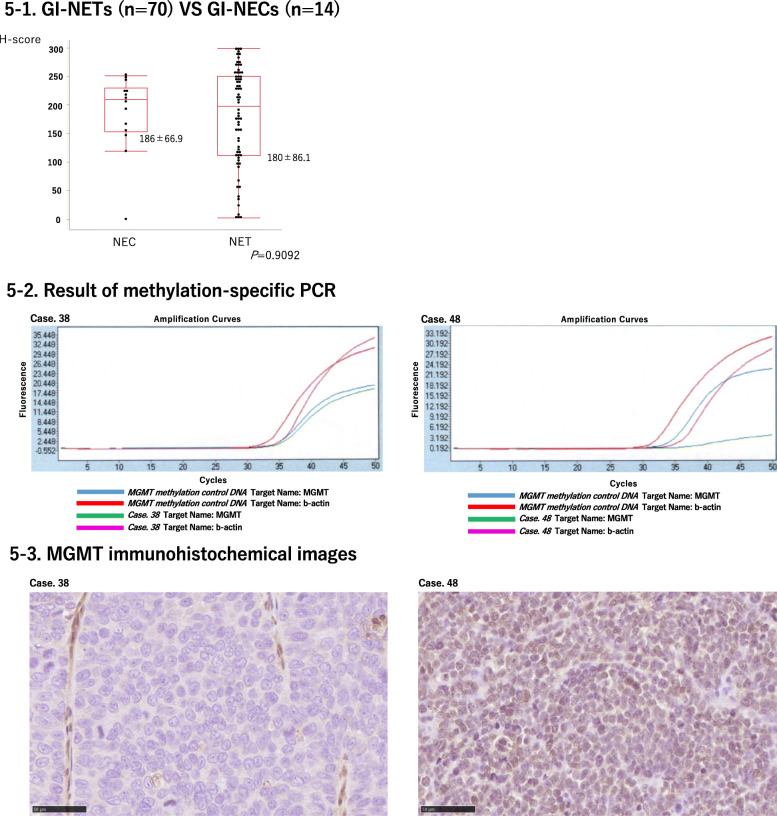
MGMT status in GI-NETs and GI-NECs. **5-1** H-scores were not significantly different between NETs and NECs. Data are expressed as mean ± SD (round the fourth digit). **5-2** MGMT promoter hypermethylation was detected in two (case 38, 48) of 14 GI-NEC patients. **5-3** Representative images of MGMT immunohistochemistry of case 38 and 48. Case 38 and 48 demonstrated MGMT score 0 and score 3, respectively

MGMT and H scores were not significantly different between NETs and NECs. Results were summarized in Table 7 and Fig. 5-1.

**Table 7:** Comparison of MGMT immunoreactivity of NETs and NECs

MGMT score	NET	NEC	*P* value
Score 0	1	1	0.1262
Score 1	15	0	
Score 2	15	4	
Score 3	39	9

### MGMT promoter methylation status

We further evaluated MGMT promoter methylation by methylation-specific PCR. Clinicopathological findings of GI-NEN cases in which MGMT promoter methylation status was analyzed using methylation-specific PCR were summarized in Table 8. MGMT promoter methylation was not detected in the 35 GI-NET cases analyzed but detected in two (case 38 and 48) of 14 GI-NEC patients. Results of methylation-specific PCR in cases 38 and 48 were presented in Fig. 5-2. Cases 38 and 48 demonstrated MGMT score 0 and score 3, respectively in its immunohistochemistry. Images of MGMT immunohistochemistry in cases 38 and 48 were illustrated in Fig. 5-3.

**Table 8:** Clinicopathological findings of GI-NEN cases in which MGMT promoter methylation status was analyzed using methylation-specific PCR

Case No.^a^	Primary site	Grade	Ki-67 labeling index	MGMT score	H-score	MGMT methylation-specific PCR
1	rectum	NET G2	6.83572	1	3.65854	negative
2	stomach	NET G2	10.9264	2	182.374	negative
3	rectum	NET G2	3.73832	3	271.554	negative
4	rectum	NET G1	1.17057	3	257.354	negative
5	rectum	NET G1	1.38648	3	207.671	negative
6	duodenum	NET G1	0.874317	3	232.395	negative
7	rectum	NET G1	0.334076	3	175.391	negative
8	duodenum	NET G2	5.42636	3	291.156	negative
9	rectum	NET G1	0.475436	3	246.119	negative
10	duodenum	NET G2	3.52588	3	176.573	negative
11	duodenum	NET G1	2	3	240.253	negative
12	duodenum	NET G1	1.97239	3	275.166	negative
13	rectum	NET G1	1.33038	3	298.588	negative
14	rectum	NET G1	0.740741	3	270.837	negative
15	rectum	NET G1	1.37741	3	243.583	negative
16	rectum	NET G2	11.9792	3	204.144	negative
17	duodenum	NET G1	2.89389	2	111.254	negative
18	rectum	NET G1	0.788955	3	243.867	negative
19	duodenum	NET G2	12.8111	2	282.641	negative
20	rectum	NET G1	1.44778	3	298.456	negative
21	duodenum	NET G1	1.0043	3	227.732	negative
22	rectum	NET G2	3.78007	3	288.949	negative
23	rectum	NET G1	0.797101	3	248.495	negative
24	stomach	NET G2	3.10786	3	260.254	negative
25	rectum	NET G1	2.23842	3	254.59	negative
26	rectum	NET G1	1.46036	3	268.528	negative
27	rectum	NET G1	1.27907	3	232.112	negative
28	stomach	NET G1	1.86757	3	217.736	negative
29	stomach	NET G2	3.7929	1	67.3792	negative
30	stomach	NET G1	1.60494	3	296.608	negative
31	rectum	NET G1	2.55924	2	191.369	negative
32	rectum	NET G1	0.840925	3	257.453	negative
33	duodenum	NET G1	1.90476	2	117.661	negative
34	rectum	NET G2	7.36722	2	141.194	negative
35	duodenum	NET G1	0.623539	1	158.554	negative
36	EG junction	NEC	96.223	3	223.134	negative
37	stomach	NEC	99.1404	2	193.032	negative
38	sigmoid colon	NEC	92.9985	0	0	positive
39	esophagus	NEC	74.7155	3	217.818	negative
40	stomach	NEC	99.7234	3	246.952	negative
41	esophagus	NEC	100	3	243.81	negative
42	stomach	NEC	77.2152	2	205.957	negative
43	stomach	NEC	85.7143	3	250.857	negative
44	stomach	NEC	79.533	2	166.723	negative
45	esophagus	NEC	99.2844	3	119.098	negative
46	rectum	NEC	95.1153	3	146.898	negative
47	rectum	NEC	88.974	2	212.279	negative
48	sigmoid colon	NEC	98.7198	3	155.372	positive
49	duodenum	NEC	98.7441	3	225.577	negative

^a^Patient characteristics of these cases: male, *n* = 32; female, *n* = 17/ Median age in years (range), 66 (39–86)

## Discussion

In this study, we examined MGMT and GLUT2 status using IHC and methylation analysis to explore their clinicopathological significance in GI-NENs. To the best of our knowledge, this is the first study that evaluated MGMT and GLUT2 immunoreactivity in GI-NENs using different analytical methods, including manual/visual analyses (MGMT and GLUT2 scores) and digital image analyses (H-score) in foregut and hindgut GI-NENs.

MGMT was reported to be expressed in 85.5% of GI-NETs [30], which is consistent with results of our present study, i.e., MGMT scores of ≥2 and ≥ 3 were 77% (54/70) and 56% (39/70), respectively. GI-NETs are generally characterized as neoplasms with relatively abundant MGMT [30]. The GLUT2 status has not been previously studied in GI-NETs. In our present study, GLUT2 scores of ≧ 4 and ≧ 6 were 30% (21/70) and 4.3% (3/70), respectively. Of particular interest, GLUT2 was not detected in 30% (21/70) of the GI-NETs studied. These results of the status of GLUT2 and MGMT status in GI NET above were consistent with results of STZ therapeutic efficacy [7, 8]. However, it is also true that in our present study, 1/37 hindgut and 4/ 33 foregut NET cases had relatively high GLUT2 and low MGMT scores (Table 3) and those cases above could be potential candidates for STZ therapy, emphasizing the importance of evaluating MGMT and GLUT2 statuses in GI-NET patients by using IHC before starting STZ treatment. However, further investigations are required to clarify MGMT and GLUT2 status as potential surrogate makers of STZ in GI-NET patients.

In the digestive system, GLUT2 is well known to be present in L-cells and enterocytes and is mainly located in the basolateral membrane of the enterocytes [19]. However, in our present study, membranous GLUT2 immunoreactivity was not detected in any of the GI-NET cases examined, regardless of histological grades and sites of their origin. In enterocytes, GLUT2 is stored in intracellular vesicles and translocated to the apical membrane when the glucose concentration in the intestinal lumen increases [18, 31], which could account for cytoplasmic localization of GLUT2 in GI-NETs.

In hindgut NETs, a significantly negative or inverse correlation was detected between the MGMT or GLUT2 status and Ki-67 LI of tumor cells. Decreased MGMT expression in tumor cells was considered to be associated with increased risks of carcinogenesis and could induce much higher tumor cell proliferation [20]. All the hindgut NET cases examined in our present study were rectal NETs. In the normal rectal mucosa, L-cells and enterochromaffin (EC) cells were reported to exist as neuroendocrine cells [19, 32], and GLUT2 was located in L-cells [19]. Therefore, relatively low GLUT2 expression levels could be explained by the deviation of the phenotypes from normal differentiation toward neuroendocrine cells or L-cells in the hindgut, and this particular deviation could be more pronounced in tumors with higher histological grades. Most foregut GI-NETs arose in the stomach and duodenum. In the normal mucosa of the stomach and duodenum, EC cells, EC-like cells, D cells, and G cells exist as neuroendocrine cells, and NET development is generally considered more complicated in the foregut than hindgut [19]; this could partly account for the lack of correlation between GLUT2 scores and Ki-67 LI in foregut GI-NETs, but would need to be clarified by further investigations.

STZ has been frequently administered to patients with metastasis or in advanced stages of NETs [33]. Therefore, it has become pivotal to evaluate the status of MGMT and GLUT2 in metastatic lesions as possible surrogate markers of STZ therapy. In this study, we examined whether MGMT and GLUT2 scores were different between primary and metastatic GI-NET lesions, although the number of metastatic cases available for examination was rather limited in our present study. However, despite this limitations, among six metastatic cases examined, four had lower GLUT2 and one lower MGMT status in the metastatic than the primary lesions. In all the cases examined, both GLUT2 and MGMT were by no means increased in metastatic lesions compared to those in primary tumors. In hindgut NET cases, two cases had lower GLUT2 and one lower MGMT status in the metastatic lesions with higher Ki-67 LI compared to those at primary tumor site. This finding was also consistent with the significant negative correlation detected between MGMT and GLUT2 status and Ki-67 LI in tumor cells. Reassessment of the Ki-67 LI in metastatic lesions has been proposed to more accurately predict clinical outcome of GI-NET patients than evaluation based on primary lesions alone [34]. Therefore, re-assessment of MGMT and GLUT2 scores in metastatic lesions, when available, could provide more clinically important information regarding the therapeutic efficacy of STZ in those metastatic lesions.

Among NET G1 cases examined, those harboring lymph node metastasis at the time of initial diagnosis had significantly lower MGMT status than those not in primary lesion. This particular correlation was not detected in GLUT2 status. These results were also consistent with the association of MGMT downregulation with progression of malignant tumors [20, 22]. In GI-NET patients, less invasive therapy such as endoscopic submucosal dissection or mucosal resection have often been administered [35]. It is therefore important to predict the clinical course of these patients, especially with respect to the status of lymph node metastasis, during histological evaluation of biopsy specimens. MGMT scoring system could therefore contribute to stratify the clinical outcome of GI-NET G1 patients.

MGMT expression has been well known to be reduced by MGMT promoter hypermethylation [20]. In neuroendocrine neoplasms of the lung, pancreas, and other sites, MGMT promoter methylation was reported in 28.4% of the cases studied [36]. In our present study, MGMT promoter hypermethylation, as evaluated by methylation-specific PCR, was not detected in any of the 35 GI-NET cases, but detected in 2/14 GI-NEC cases studied. Therefore, among the patients with neuroendocrine neoplasms (NENs), MGMT promoter methylation could be detected more frequently in high grade NENs than low or intermediate grade NENs. However, there were no significant differences in MGMT immunoreactivity, obtained by both manual (MGMT score system) and digital analysis (H-score system), between GI-NETs and GI-NECs. In addition, of the two MS-PCR positive cases, one was immunohistochemically negative (MGMT score 0), but the other positive or MGMT score of 3. Discrepancy between MGMT IHC and methylation-specific PCR results has been often reported in glioblastomas and pancreatic NETs [16, 23, 27], which is also consistent with our present results. This discrepancy is generally considered to reflect the heterogeneity of MGMT and/or the regulation of MGMT expression by factors other than methylation in the promoter region [16]. In addition, the accuracy of CpG island methylation using methylation specific PCR alone was considered lower than that of the bisulfite sequence and the total expression of MGMT itself is not necessarily regulated only by its methylation [16]. Therefore, immunohistochemical analysis of MGMT could provide important information on its expression regardless of its methylation status but further investigations are required for clarification.

## Conclusions

We examined GLUT2 and MGMT status, which could influence therapeutic efficacy of STZ, in GI-NETs according to embryological classification and grades. In hindgut-NETs, both MGMT and GLUT2 tended to decrease as grades or Ki-67 LI increased, but no such correlation was detected in foregut NETs. However, in hindgut-NETs examined, some cases demonstrated discrepancy between MGMT and GLUT2 status and it would be important to evaluate MGMT and GLUT2 status in tumor cells before administering STZ in GI-NET patients. In addition, MGMT status of tumor cells could also serve as a prognostic indicator of GI-NET G1, independent of the Ki-67 LI.

## Supplementary Information

**Additional file 1.**

**Additional file 2.**

**Additional file 3.**

**Additional file 4.**

**Additional file 5.**

## Data Availability

The datasets used and/or analyzed during the current study are available from the corresponding author on reasonable request. The dataset(s) supporting the conclusions of this article is (are) included within the article (and its additional file(s)).

## Abbreviations

STZ: Streptozocin
PanNET: Pancreatic neuroendocrine tumor
GI-NET: Gastrointestinal neuroendocrine tumor
NEN: Neuroendocrine neoplasm
NEC: Neuroendocrine carcinoma
MGMT: *O*^6^-methylguanine DNA methyltransferase
GLUT2: Glucose transporter 2
EC cell: Enterochromaffin cell
ECL cell: Enterochromaffin like cell
