# Supplementary material for: O6-methylguanine DNA methyltransferase and glucose transporter 2 in foregut and hindgut gastrointestinal neuroendocrine neoplasms
Source: BMC Cancer. 2020 Dec 7;20:1195. doi: 10.1186/s12885-020-07579-6 (PMC7720403; doi:10.1186/s12885-020-07579-6)
Supplement: Supplementary file 1 — Additional file 1. [file 12885_2020_7579_MOESM1_ESM.pdf]

Positive Control of GLUT2 and MGMT

GLUT2

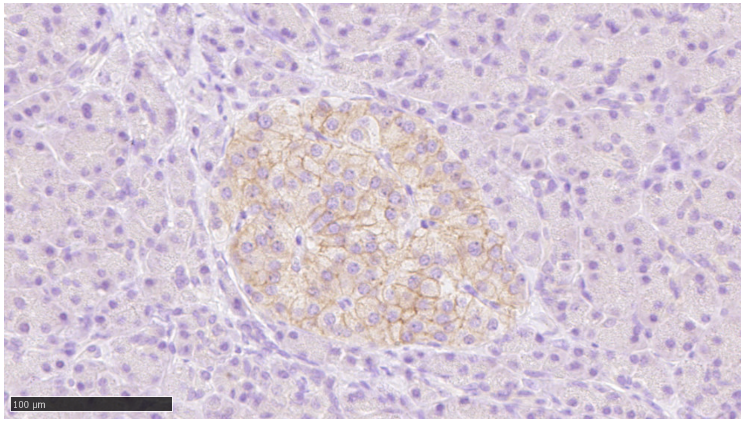

Membranous immunoreactivity on islet of langerhans

MGMT

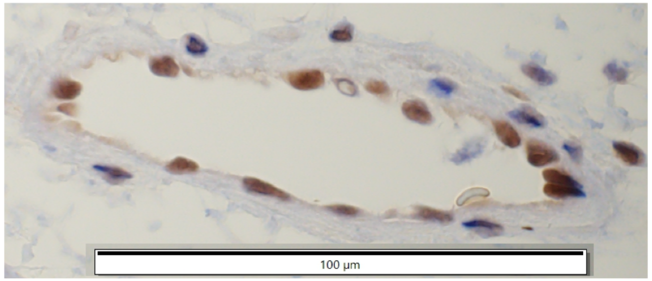

Nucleic immunoreactivity on vascular endothelial cell
