# Supplementary material for: O6-methylguanine DNA methyltransferase and glucose transporter 2 in foregut and hindgut gastrointestinal neuroendocrine neoplasms
Source: BMC Cancer. 2020 Dec 7;20:1195. doi: 10.1186/s12885-020-07579-6 (PMC7720403; doi:10.1186/s12885-020-07579-6)
Supplement: Supplementary file 2 — Additional file 2. [file 12885_2020_7579_MOESM2_ESM.pdf]

HALO image analysis software with the CytoNuclear IHC v1.6 algorithm module

Step 1 Marking tumoral area

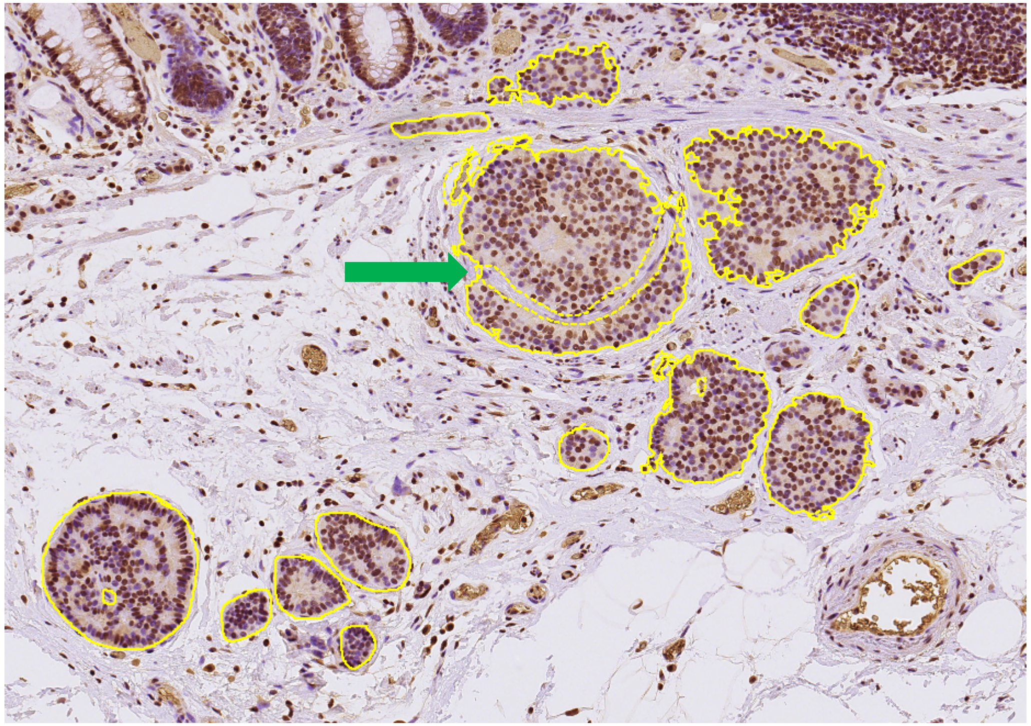

Marking tumor (yellow line) and removing interstitium including vessels (yellow broken line, see green arrow)

Step 2 Setting “Stain Selection”

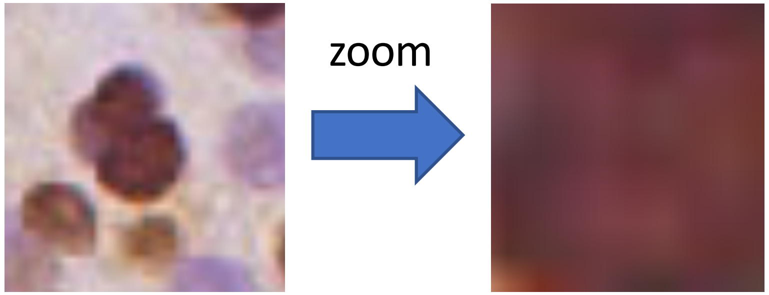

Selecting representative stained cell, adjust average intensity by regulate pixel zoom and setting “Positive Stain”
