# Supplementary material for: O6-methylguanine DNA methyltransferase and glucose transporter 2 in foregut and hindgut gastrointestinal neuroendocrine neoplasms
Source: BMC Cancer. 2020 Dec 7;20:1195. doi: 10.1186/s12885-020-07579-6 (PMC7720403; doi:10.1186/s12885-020-07579-6)
Supplement: Supplementary file 3 — Additional file 3. [file 12885_2020_7579_MOESM3_ESM.pdf]

# HALO image analysis software with the CytoNuclear IHC v1.6 algorithm module

## Step 3 Setting parameter of "Cell Detection"

***Cell Detection***

Nuclear Contrast Threshold

Minimum Nuclear OD

Nuclear size

Minimum Nuclear Roundness

Nuclear Segmentation Aggressiveness

Setting parameter of nuclear contrast, optical density, size, and shape by regulating above parameters

## Step 4 Setting the thresholds of immunoreactivity

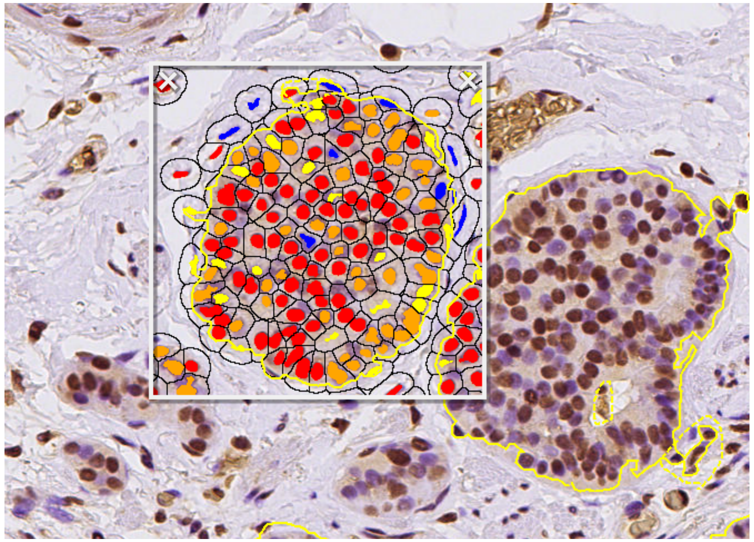

Blue: negative
Yellow: weak positive
Orange: moderate positive
Red: strong positive

Setting thresholds of negative, weak, moderate and strong positive
