# Supplementary material for: O6-methylguanine DNA methyltransferase and glucose transporter 2 in foregut and hindgut gastrointestinal neuroendocrine neoplasms
Source: BMC Cancer. 2020 Dec 7;20:1195. doi: 10.1186/s12885-020-07579-6 (PMC7720403; doi:10.1186/s12885-020-07579-6)
Supplement: Supplementary file 4 — Additional file 4. [file 12885_2020_7579_MOESM4_ESM.pdf]

HALO image analysis software with the CytoNuclear IHC v1.6 algorithm module

Step 5 Analyzing the all marked tumor area and confirming the Results

Results

% Stain 1 Positive Cells

% Stain 1 0+ Cells

% Stain 1 1+ Cells

% Stain 1 2+ Cells

% Stain 1 3+ Cells

Stain 1 H-score

% Stain 0+: negative
% Stain 1+: weak positive
% Stain 2+: moderate positive
% Stain 3+: strong positive

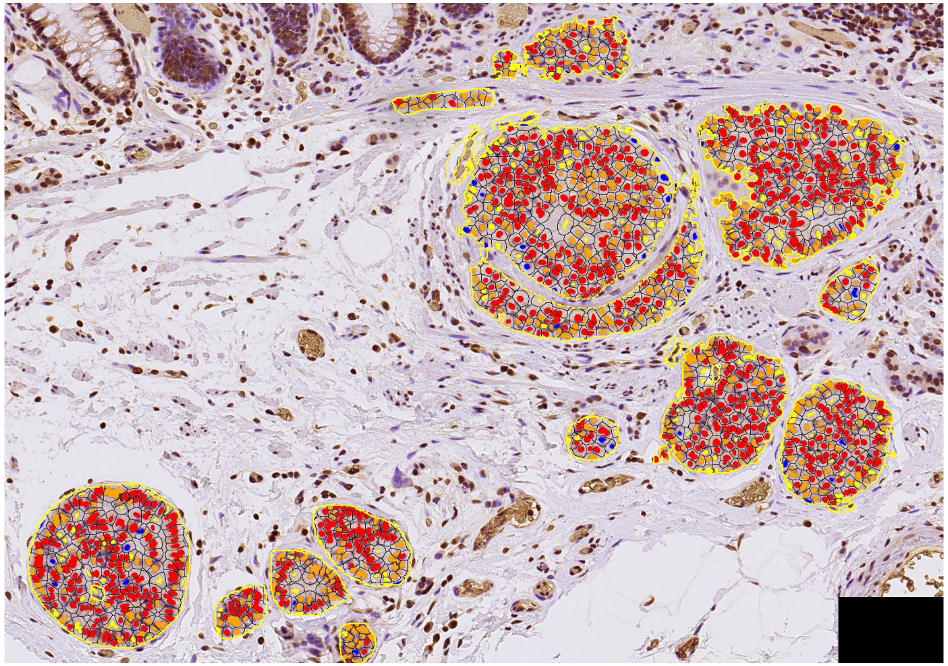

Blue: negative
Yellow: weak positive
Orange: moderate positive
Red: strong positive
